# Use of Dual Orexin Receptor Antagonists for the Prevention of Delirium: A Systematic Review and Meta‐analysis

**DOI:** 10.1002/alz70857_103085

**Published:** 2025-12-25

**Authors:** Arianna R Paolone, Shehraz Riar, Shajjan Vejayavarnan, Justin Y Lee

**Affiliations:** ^1^ Royal College of Surgeons in Ireland, Dublin, Dublin, Ireland; ^2^ McMaster University, Hamilton, ON, Canada; ^3^ University of Limerick, Limerick, Limerick, Ireland; ^4^ Centre for Integrated Care, St. Joseph's Health System, Hamilton, ON, Canada; ^5^ Geras Centre for Aging Research, Hamilton, ON, Canada

## Abstract

**Background:**

Delirium and dementia are leading causes of cognitive impairment in older adults and have a complex and interconnected relationship. Delirium can exacerbate cognitive decline in dementia, but it is also an independent risk factor for the development of long‐term cognitive impairment and new‐onset dementia. Although many medications have been evaluated, studies have not shown any medication to be effective for the prevention or treatment of delirium. Their use also carry a high risk of adverse effects where the balance of benefits and harms is unclear in older adults and those with dementia. Dual orexin receptor antagonists (DORAs) have emerged as a new, potentially safer class of medications for the management of delirium. We conducted a systematic review to assess the effects of DORAs on the prevention and treatment of delirium

**Methods:**

We searched MEDLINE, EMBASE, and CENTRAL for primary studies evaluating the use of DORAs for delirium prevention or treatment compared to non‐users. Citation screening, data abstraction, risk of bias, and quality of evidence assessments were conducted in duplicate. Random‐effects model meta‐analyses were conducted.

**Results:**

Three randomized controlled trials (345 participants) and 6 observational studies (1,943 participants) met inclusion criteria. In pooled meta‐analysis, DORA use led to a clinically and statistically significant decrease in the overall incidence of delirium (OR 0.28, 95% CI 0.18–0.42). DORAs decreased the odds of delirium in both post‐operative patients (OR 0.24, 95% CI 0.07–0.83) and intensive care unit patients (OR 0.28, 95% CI 0.18–0.45).

**Discussion:**

The preventative effect of DORAs on delirium was consistently observed in different study designs (RCTs vs observational), different clinical settings (post‐operative vs. ICU) and different DORA agents. The magnitude of effect appears to be both statistically and clinically significant and may represent an important intervention to reduce the impact of delirium on long‐term cognitive impairment and decline. However, data on their use in older adults, patients living with dementia and the treatment of delirium is limited.

**Conclusion:**

DORAs appear to reduce the incidence of delirium in hospitalized patients, but additional research is required to evaluate their effects in older adults and those living with dementia.

